# Gene signatures and prognostic values of m1A-related regulatory genes in hepatocellular carcinoma

**DOI:** 10.1038/s41598-020-72178-1

**Published:** 2020-09-15

**Authors:** Qingmiao Shi, Chen Xue, Xin Yuan, Yuting He, Zujiang Yu

**Affiliations:** 1 Gene Hospital of Henan Province, Precision Medicine Center, The First Affiliated Hospital of Zhengzhou University, Zhengzhou, 450052 Henan People’s Republic of China; 2 Department of Infectious Diseases, The First Affiliated Hospital of Zhengzhou University, Zhengzhou, 450052 Henan People’s Republic of China

**Keywords:** Cancer epidemiology, Gene ontology

## Abstract

Hepatocellular carcinoma (HCC) ranks fourth in cancer-related mortality worldwide. N1-methyladenosine (m1A), a methylation modification on RNA, is gaining attention for its role across diverse biological processes. However, m1A-related regulatory genes expression, its relationship with clinical prognosis, and its role in HCC remain unclear. In this study, we utilized The Cancer Genome Atlas-Liver Hepatocellular Carcinoma (TCGA-LIHC) database to investigate alterations within 10 m1A-related regulatory genes and observed a high mutation frequency (23/363). Cox regression analysis and least absolute shrinkage and selection operator were used to explore the association between m1A-related regulatory genes expression and HCC patient survival and identified four regulators that were remarkably associated with HCC patient prognosis. Additionally, an independent cohort from International Cancer Genome Consortium was studied to validate our discoveries and found to be consistent with those in the TCGA dataset. In terms of mechanism, gene set enrichment analysis linked these four genes with various physiological roles in cell division, the MYC pathway, protein metabolism, and mitosis. Kyoto Encyclopedia of Genes and Genomes analysis revealed that PI3K/Akt signaling pathway had potential relevance to m1A-related regulatory genes in HCC. These findings indicate that m1A-related regulatory genes may play crucial roles in regulating HCC progression and be exploited for diagnostic and prognostic purposes.

## Introduction

Hepatocellular carcinoma (HCC) ranks fourth in cancer-related deaths worldwide^1^. Risk factors for HCC mainly consist of chronic viral hepatitis, alcoholism, and metabolic disorders^2–4^. HCC treatments have improved in recent years, with liver resection, transplantation, ablation, transarterial embolization, and systemic pharmacological treatment showing some efficacy in improving overall survival^5–7^. However, the majority of HCC patients miss their optimal treatment window due to late diagnosis^8^. Therefore, to improve the currently dismal outcomes of HCC patients, it is critical to identify novel biomarkers for early HCC detection and prognostication.

The dynamic and reversible chemical modification of RNA plays a vital role in post-transcriptional gene regulation^9^. Over 100 post-transcriptional RNA modifications, including N6-methyladenosine (m6A), 5-methylcytidine (m5C), N1-methyladenosine (m1A), and pseudouridine, have been found to have important functions in cellular differentiation, protein production, and biological regulation^10–14^. Despite m1A being discovered five decades ago^15^, progress in characterizing its role of modifying RNA has only happened recently^11^. m1A is produced by attaching a methyl group to the N1 position of adenosine^16^. Because of the methyl group and a positive charge in m1A, it is capable of markedly altering RNA structure and the strength of protein-RNA interactions. m1A is enriched within 5′ untranslated regions (UTRs) and the upstream vicinity of start codons, has been shown to actively respond to physiological conditions, and correlates positively with protein production^11,17^.

RNA modifications are dynamically regulated at the post-transcriptional level^9^. m1A methylation regulators are composed of “writers” (TRMT10C, TRMT61B, TRMT6, TRMT61A), “erasers” (ALKBH1, ALKBH3), and “readers” (YTHDF1, YTHDF2, YTHDF3, YTHDC1)^18–21^. The “writers” comprise a methyltransferase complex which deposits the m1A mark. Although TRMT61B mainly localizes to mitochondria and forms a homo-oligomer^20^, TRMT61A and TRMT6 form α2β2 heterotetramers to create the cytoplasmic tRNA m1A methyltransferase. The “erasers” act as m1A demethylases to remove methyl groups from m1A and make its function reversible^19^. The “readers” decode m1A methylation marks and mediate the downstream effects of post-transcriptional regulation^18^. In general, “writers” and “erasers” determine the prevalence and distribution of m1A, whereas “readers” mediate m1A-related functions.

m1A dysregulation affects multiple biological processes, including cell proliferation, self-renewal programs, and apoptosis. Research has shown that high expression of hTrm6p/hTrm61p m1A transmethylase was correlated with m1A levels in urine and the occurrence of bladder cancer^22^. Another study demonstrated that m1A-related regulatory genes were dysregulated in gastrointestinal cancer and associated with mTOR and ErbB pathways^23^. In addition, the alteration of DNA methylation was described in HCC and may act vital roles in tumorigenesis^24,25^. However, the expression and functional roles of m1A-related regulatory genes in HCC remain largely unknown.

In this study, using HCC data from The Cancer Genome Atlas-Liver Hepatocellular Carcinoma (TCGA-LIHC) database, we analyzed clinical and sequencing data, the alterations, and relationships between ten m1A-related regulatory genes and clinicopathological features, and the impact of genetic alterations on survival. Four m1A-related regulator genes selected form least absolute shrinkage and selection operator (LASSO) analysis were identified as important HCC target molecules and verified in an independent validation dataset from International Cancer Genome Consortium-Liver Hepatocellular Carcinoma (ICGC-LIHC-JP), which provided rationale for studying the relationship between m1A expression and HCC prognosis. In addition, gene set enrichment analysis (GSEA) was conducted to explore the roles of these differentially expressed genes. Kyoto Encyclopedia of Genes and Genomes (KEGG) analysis was used to seek potential signaling pathway. These findings indicate that m1A-related regulatory genes perform crucial functions in regulating the progression of human HCC.

## Results

### Alterations of m1A-related regulatory genes in HCC samples

**Figure 1:**
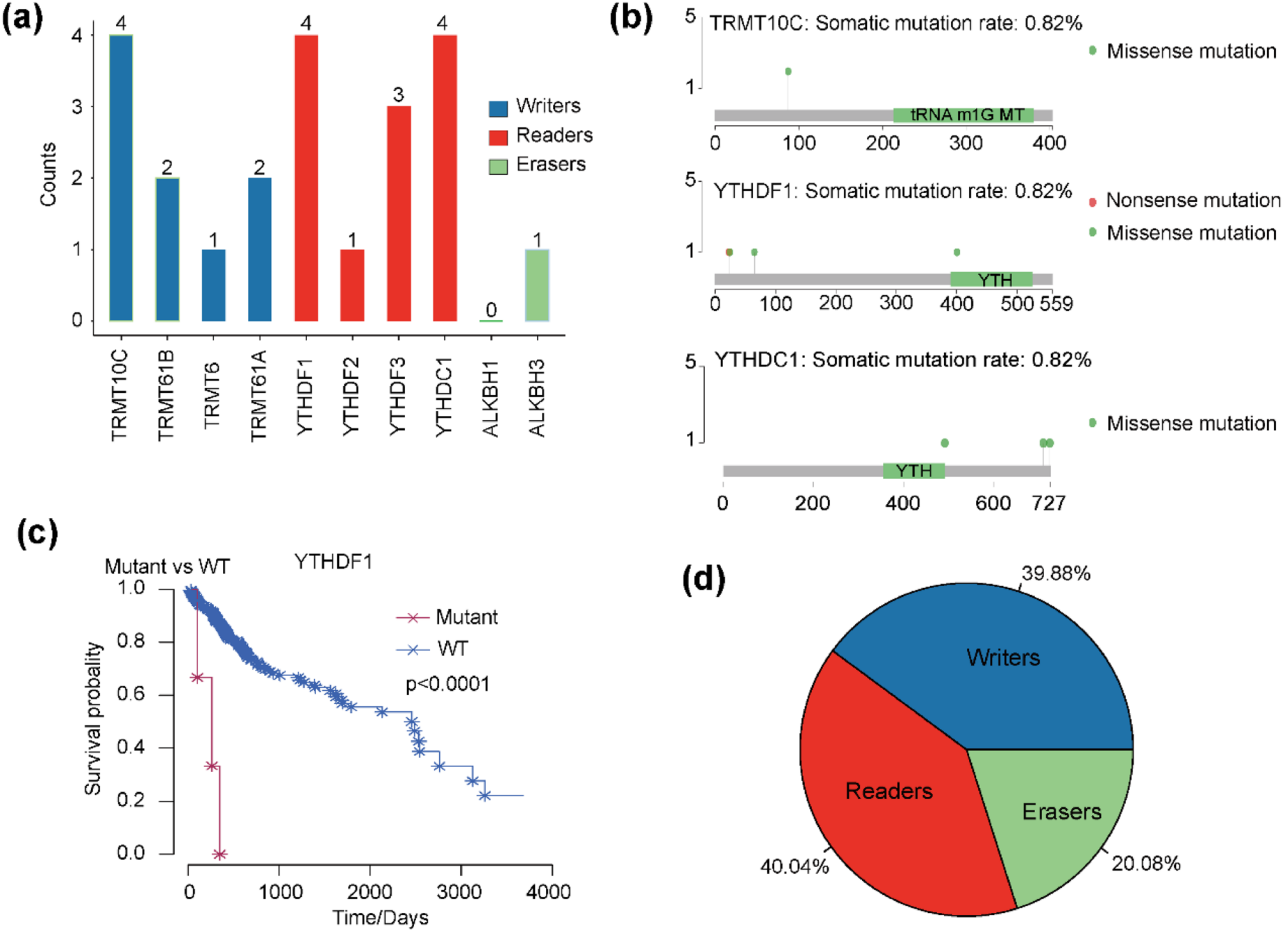
Alterations of m1A-related regulatory genes in HCC patients. (**a**) Mutation counts for m1A-related regulatory genes in 364 HCC patients. (**b**) The mutation sites of TRMT10C, YTHDF1, and YTHDC1. (**c**) Kaplan–Meier curve of survival probability of HCC patients with YTHDF1 gene mutation. (**d**) The CNV percentage of different m1A regulator subtypes.

To investigate alterations to m1A-related regulatory genes in HCC samples, especially mutations, single nucleotide variation (SNV) and copy number variation (CNV), we analyzed 364 patients with SNV data and 376 patients with CNV data extracted from TCGA. m1A-related regulatory gene mutations were discovered in 20 independent samples (Table S1). Among them, the “reader” genes YTHDC1 and YTHDF1 and “writer” gene TRMT10C had the highest mutation counts (Fig. 1a). The mutation frequency of “eraser” genes was lower than “writer” and “reader” genes. In terms of SNV function, annotated functional changes of m1A-related regulatory genes occurred in 12 samples (Table S2). In addition, seven out of ten m1A-related regulatory genes possessing functional changes, six of which had missense mutations (Table S3).

TRMT10C (‘writer’), YTHDF1 (‘reader’) and YTHDC1 (‘reader’) genes had the greatest number of mutations, with missense mutations potentially leading to impaired function (Fig. 1b), affecting m1A signaling in cells and causing physiological disorder in tumour cells. Using the seven functionally-altered genes to predict HCC patient survival, we found that HCC samples with TRMT10C, YTHDF1 and YTHDC1 gene mutations had worse prognosis than samples without mutations. In particular, there was a significant correlation between YTHDF1 gene mutation and prognosis (Fig. 1c). Additionally, CNV for the ten m1A-related regulatory genes were frequent in the 376 HCC samples (Fig. 1d). The highest frequency of CNV events occurred in the m1A “reader” gene YTHDF3 (53.97%), followed by YTHDC1 (34.29%), while the “writer” gene TRMT10C had the lowest frequency (13%) (Table S4). These findings suggest that alterations to m1A-related regulatory genes are relatively common in HCC.

### Correlation between m1A-related regulatory gene alterations and clinicopathological features

Given the role of m1A-related regulatory genes in carcinoma progression, we next evaluated the correlation between alterations to m1A-related regulatory genes and patient clinicopathological features. HCC patient survival was significantly correlated with higher tumour stage (T stage) and TNM stage, but not with SNV or CNV presence (Table 1). We speculate that m1A-related regulatory gene alterations may be related to changes in other disease-causing molecules in HCC. Since TP53, NQO1, and EPHX1 play critical roles in HCC pathogenesis^26–28^, we assessed the relationship between m1A-related regulatory gene alterations and changes to genes encoding these proteins. Mutations to m1A-related regulatory genes were not significantly correlated with NQO1 and EPHX1, but prominently associated with alterations in TP53. Besides, in 82/89 patients with TP53 alterations, TP53 mutations and m1A-related regulatory gene mutations were coexistent (Table 2). Taken together, not only alterations to m1A-related regulatory genes were correlated with T stage and TNM Stage, they were also found to significantly associate with TP53 mutations.

**Table1:** Cox regression analysis of clinical features and m1A-related regulatory gene alterations.

Features	beta	HR (95% CI)	wald.test	*P*
T stage	1	2.8 (1.9–4.2)	28	1.60E−07
TNM stage	1	2.8 (1.9–4.1)	27	1.80E−07
M stage	0.42	1.5 (0.98–2.3)	3.5	0.062
CNV	0.39	1.5 (0.88–2.5)	2.1	0.14
N stage	0.31	1.4 (0.88–2.1)	2	0.16
Grade	0.079	1.1 (0.73–1.6)	0.15	0.69
SNV	0.2	1.2 (0.53–2.8)	0.22	0.64

**Table 2:** The relationship between m1A-related regulatory gene alterations and three disease-causing molecules genetic alternations.

Molecule	Number	Type	Without SNV and CNV	With SNV and CNV	*X*^2^	*P*
TP53		wt	50	176	7.823775	0.005156
	n = 315	Alternation	7	82		
NQO1		wt	57	257	0.688999	0.406505
	n = 315	Alternation	0	1		
EPHX1		wt	79	233	0.114086	0.73554
	n = 315	Alternation	0	3		

### Relationship between mRNA expression of m1A-related regulatory genes and CNV patterns

**Figure 2:**
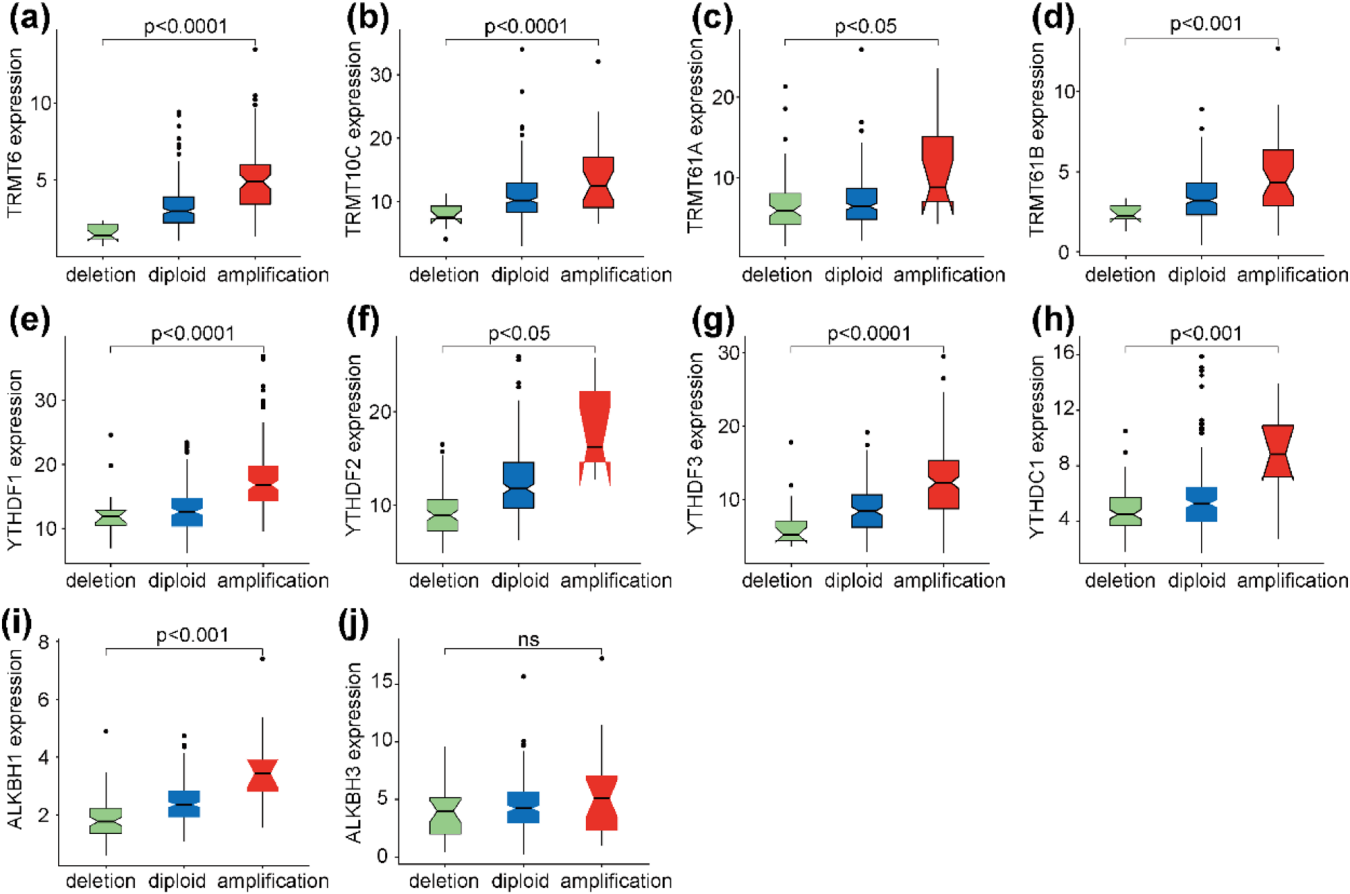
The relationship between different CNV patterns and mRNA expression levels of m1A-related regulatory genes. (**a**–**i**) The increased copy numbers of (**a**) TRMT6, (**b**) TRMT10C, (**c**) TRMT61A, (**d**) TRMT61B, (**e**) YTHDF1, (**f**) YTHDF2, (**g**) YTHDF3, (**h**) YTHDC1, and (**i**) ALKBH1 are significantly related to their mRNA expression. (**j**) CNV for ALKBH3 is not associated with its mRNA expression.

Next, we investigated the impact of m1A-related regulatory gene CNV with their mRNA expression. Because CNV alterations can affect gene expression levels via dose compensation effects, we analyzed the correlation between m1A-related regulatory gene mRNA expression levels and CNV patterns. Distinct associations were observed between mRNA expression levels and CNV patterns in 315 HCC samples. For the ten regulatory genes, increased copy numbers for nine genes were correlated with higher mRNA expression, while deletions led to decreased mRNA expression (Fig. 2a–i). Only one gene lacked a significant relationship between CNV and mRNA expression (Fig. 2j). The involvement of these nine genes throughout all m1A regulatory processes supports the notion that m1A-related regulatory gene CNV plays an essential role in m1A regulation.

### Relationship between m1A-related regulatory gene expression and prognosis in different clinical stages

**Figure 3:**
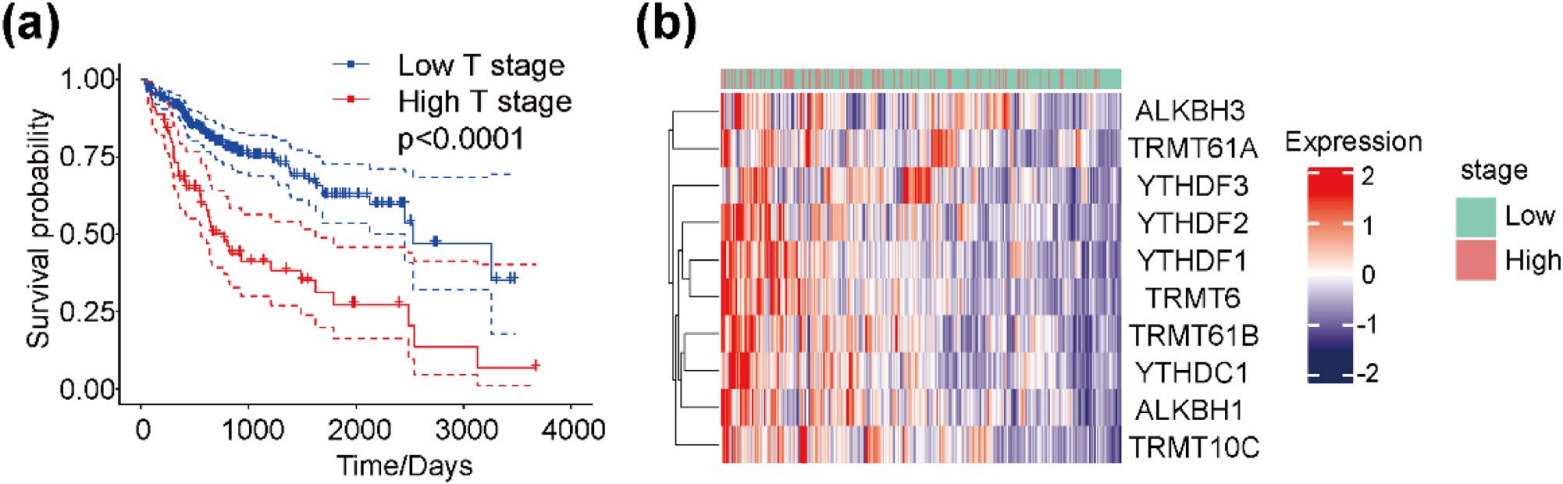
Prognosis and expression differences of m1A-related regulatory genes in different clinical stages. (**a**) Kaplan–Meier survival probability curve of different clinical T stages and patient prognoses. (**b**) Cluster heatmap of m1A-related regulatory genes and different TNM stages.

We next defined clinical stages as follows: the TNM stages I/II as low TNM stage and the TNM Stages III/IV as high TNM stage. The T1/T2 stages as low T stage and the T3/T4 stages as high T stage. Combined with the above analysis, Kaplan–Meier survival curves showed a significant correlation between T stage and HCC patient prognosis (Fig. 3a). According to the above classification, clustering of m1A-related regulatory gene expression at different TNM stages indicated that m1A-related regulatory genes tend to be highly expressed at high clinical stages (Fig. 3b).

**Figure 4:**
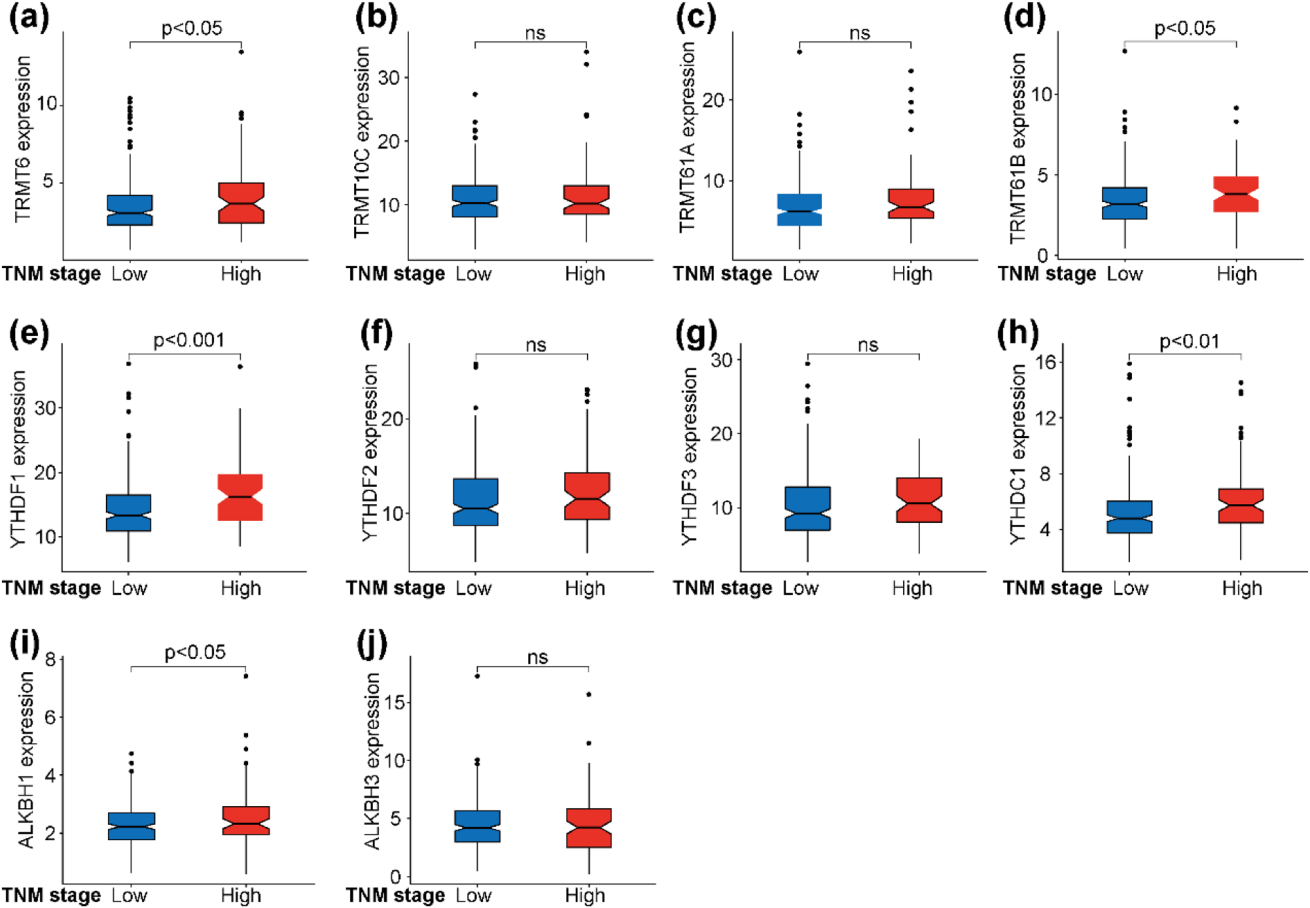
Expression differences of m1A-related regulatory genes in different clinical stages. The expression of (**a**) TRMT6, (**d**) TRMT61B, (**e**) YTHDF1, (**h**) YTHDC1, and (**i**) ALKBH1 significantly correlated with clinical TNM stages. The expression of (**b**) TRMT10C, (**c**) TRMT61A, (**f**) YTHDF2, (**g**) YTHDF3, and (**j**) ALKBH3 have no significant differences in different clinical TNM stages.

We then analyzed the expression differences of m1A-related regulatory genes at different clinical TNM stages. A significantly positive correlation between the expression of 5/10 m1A-related regulatory genes and clinical TNM stages was found (Fig. 4a–j). Among them, YTHDF1 and YTHDC1 not only had the highest mutation rate, but also a positive correlation with CNV changes and expression level. Collectively, m1A-related regulatory gene expression was significantly correlated with clinical TNM stage and prognosis.

### Prognostic value of m1A-related regulatory genes expression in HCC patients

**Figure 5:**
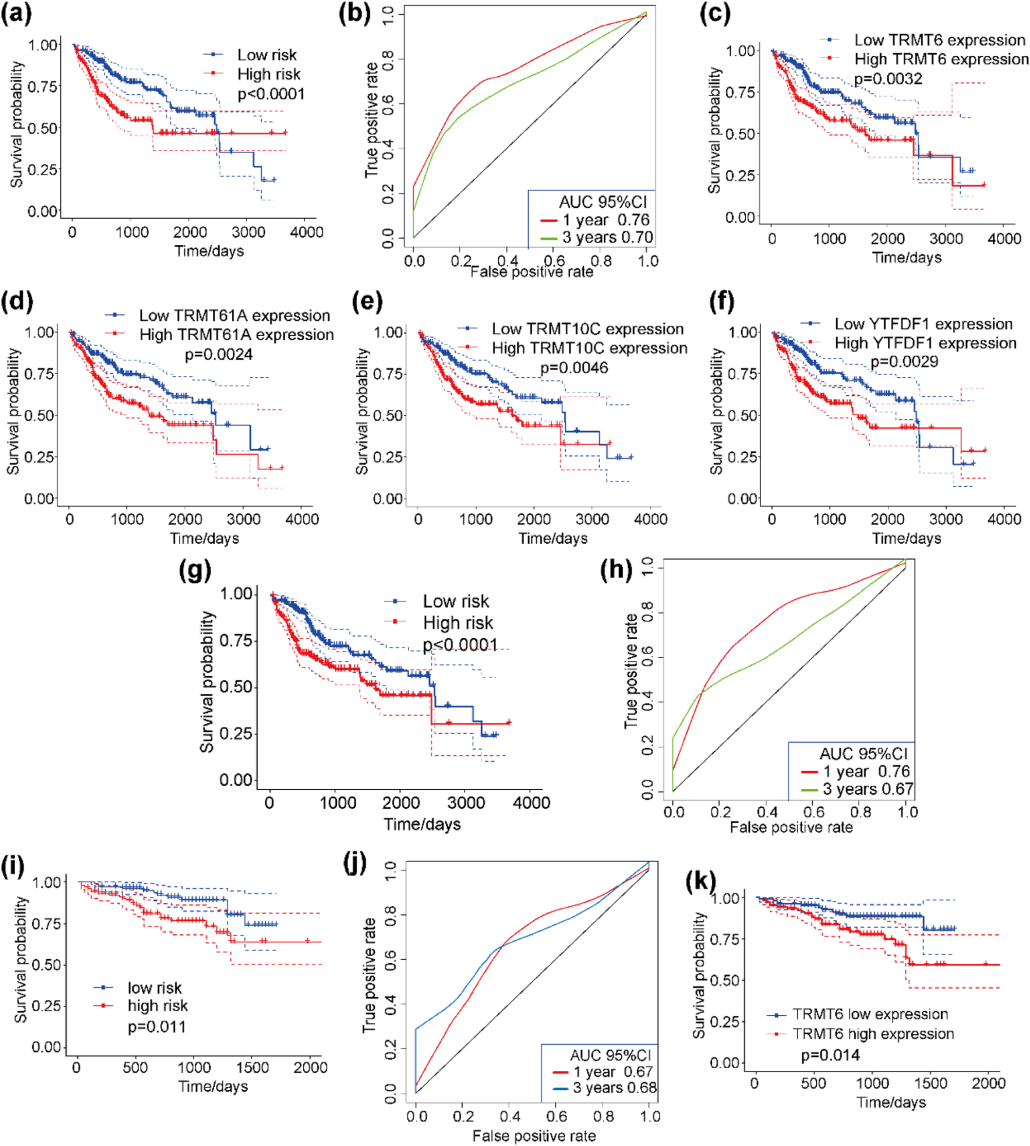
Prognostic values of m1A-related regulatory genes in HCC patients. (**a**) Kaplan–Meier survival curves of HCC patients from TCGA dataset based on 10 m1A-related regulatory genes. (**b**) Time-dependent ROC curve of 1-year and 3-year survival rate. (**c**–**f**) High expression of (**c**) TRMT6, (**d**) TRMT61A, (**e**) TRMT10C, and (**f**) YTHDF1 are associated with poor prognosis of HCC patients. (**g**) Kaplan–Meier survival curves of HCC patients from TCGA dataset based on four m1A-related regulatory genes. (**h**) Time-dependent ROC curve of 1-year and 3-year survival rate. (**i**) Kaplan–Meier survival curves of HCC patients from ICGC dataset based on four m1A-related regulatory genes. (**j**) Time-dependent ROC curve of 1-year and 3-year survival rate. (**k**) In validation dataset, high expression of the TRMT6 gene was associated with poor prognosis (*P* = 0.014).

We used univariate Cox regression analysis to explore the relationship between m1A-related regulatory gene expression and patient prognosis. As shown in Table S5, the expression levels of seven genes were significantly correlated their CNV changes and patient prognosis (*p* < 0.05). Furthermore, we also explored the effect of the 10 m1A-related regulatory genes expression on patient survival by multivariate Cox regression. A risk score based on regression coefficients from above multivariate Cox regression model was calculated to stratify patients into high-risk group and low-risk group according to median risk value. Kaplan–Meier curve showed the survival probability of high-risk group was significant decreased than low-risk group (Fig. 5a), with the areas under the curve (AUC) at 1 and 3 years being greater than 0.69 (Fig. 5b). These results indicated that m1A-related regulatory genes expression could be used as a prognostic biomarker for HCC.

### Four m1A-related regulatory genes can effectively predict the survival of HCC patients

Based on the above results, to further clarify prognostic potential, we performed LASSO analysis on the 10 m1A-related regulatory genes. Four genes TRMT6, TRMT61A, TRMT10C, and YTHDF1 were selected based on the results of 1,000 times LASSO regressions. We next used these four genes to analyze the relationship between their gene expression and patient prognosis. The results showed that high expression levels for these four genes were associated with poor patient prognosis (Fig. 5c–f). To calculate patient risk score, multivariate Cox regression analysis was conducted using these four genes. A four genes-based risk score was calculated on account of the multivariate Cox regression analysis coefficient and its expression level. Using median risk score to divide patients into high-risk and low-risk group, Kaplan–Meier curve displayed that the risk value could effectively predict HCC patient survival (*p* < 0.0001) (Fig. 5g). A risk prediction model constructed using these four m1A-related regulatory genes found that the AUC at 1 and 3 years was greater than 0.66 (Fig. 5h).

Furthermore, we used the validation dataset ICGC-LIHC-JP to analyze the relationship between the expression of the four genes above and patient survival. Based on multivariate Cox regression analysis, the four genes showed good risk prediction ability for HCC samples (*p* = 0.011) (Fig. 5i), with AUC greater than 0.67 (Fig. 5j). This dataset also confirmed that high expression of TRMT6 was associated with poor prognosis (*p* = 0.014) (Fig. 5k). These results suggest that the expression levels of these four genes have important clinical relevance to HCC patients and possess potential to be crucial HCC target molecules.

### Signaling pathway regulated by m1A-related regulatory genes

**Figure 6:**
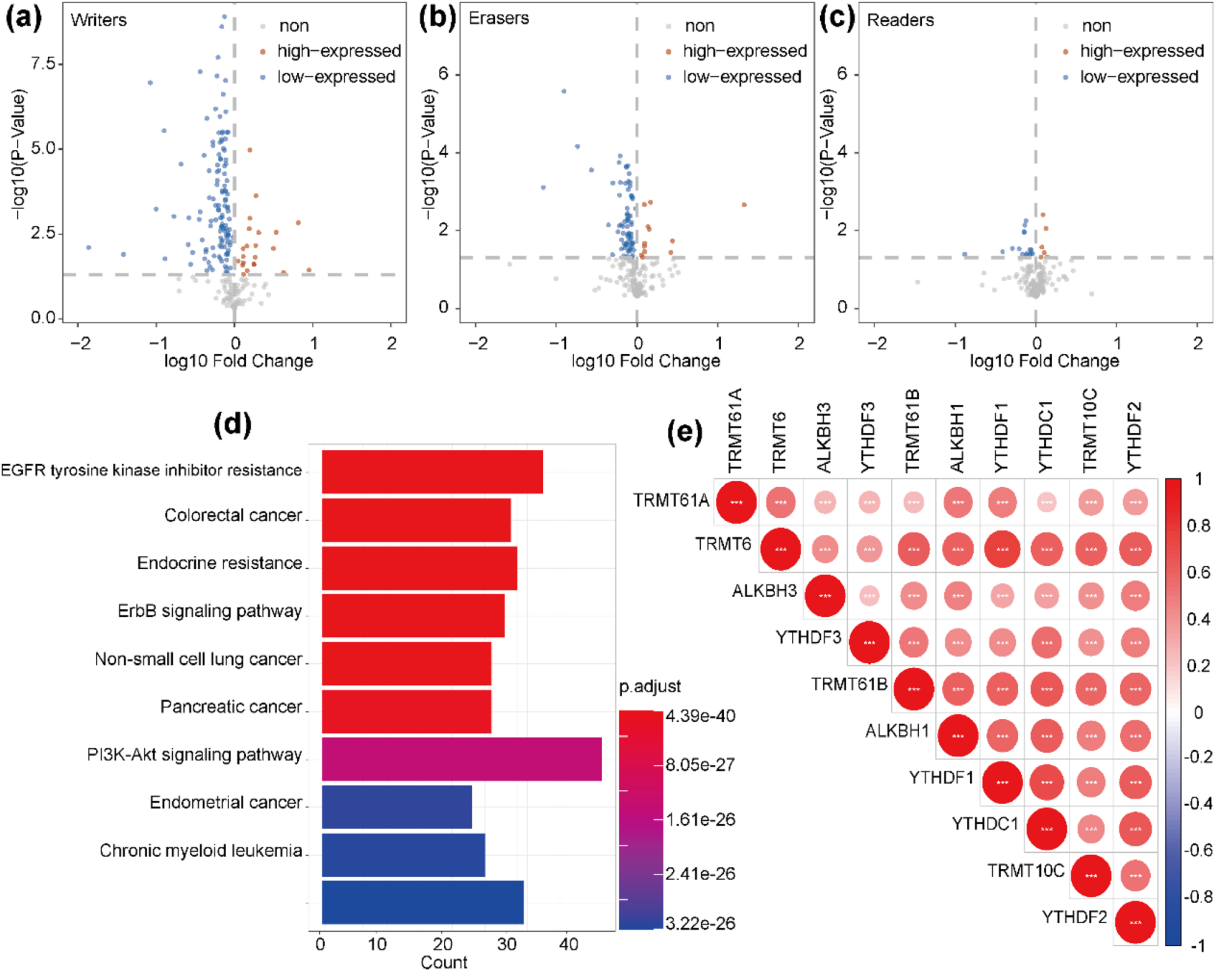
A prediction of potential pathway regulated by m1A-related regulatory genes. (**a**–**c**) The targeted proteins of m1A-related regulatory genes were differentially expressed within the cluster of “writers”, “erasers”, and “readers”. (**d**) KEGG pathway analysis of the protein coding genes. Only the top 10 KEGG pathway terms were presented. (**e**) Spearman correlation analysis of the 10 m1A-related regulatory genes.

Next, correlational analysis of protein expression was performed to explore m1A-related pathway. Adopting the cBio Cancer Genomics Portal (cBioPortal) with the Reverse Phase Protein Arrays (RPPA) data, we predicted the target proteins of m1A-related regulatory genes. Following, we analyzed the expression level of target proteins between the high and low expression groups of m1A-related regulatory genes. The differentially expressed proteins were distinguished in the cluster of “writers”, “erasers”, and “readers”, respectively (Fig. 6a–c). Later, we mapped the genes that encoding these differentially expressed proteins. KEGG pathway analysis was conducted to determine the signaling pathways related to protein coding genes. It identified 148 types of significant KEGG pathways (p_adjust_ < 0.05), especially PI3K-Akt signaling pathway (Fig. 6d). Furthermore, we researched the relevance among the ten m1A-related regulatory genes in order to have a better understanding of their interaction. It’s found the expression of YTHDF1 was significantly associated with TRMT6 in HCC (Fig. 6e).

### Functional annotation of m1A-related regulatory genes by gene set enrichment analysis

**Figure 7:**
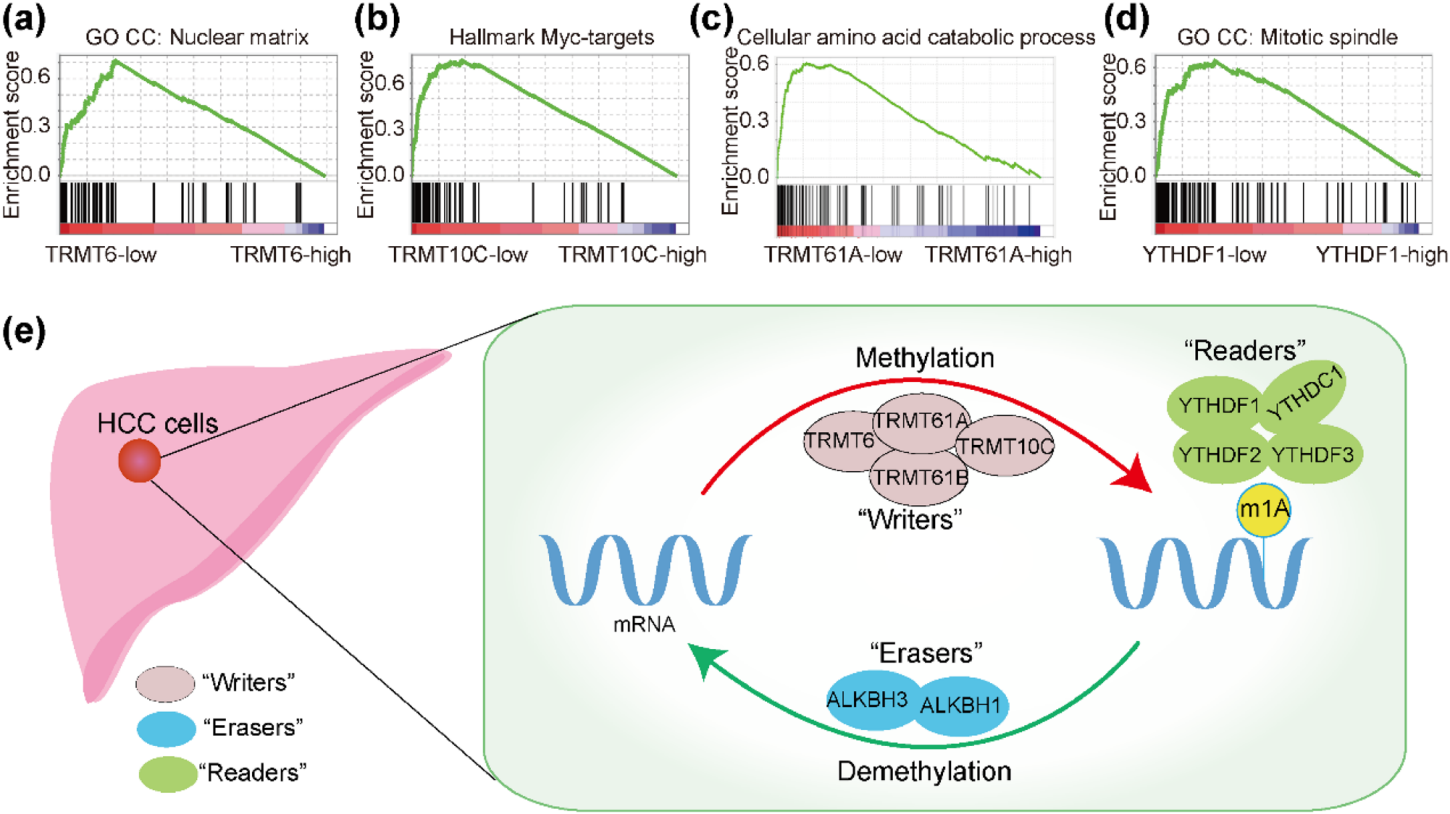
Functional annotation of m1A-related regulatory genes in HCC. (**a**–**d**) GSEA results for TRMT6, TRMT61A, TRMT10C, and YTHDF1. (**e**) A schematic of m1A-related regulatory genes. m1A is deposited on mRNAs by “writers”, removed by “erasers”, and interpreted by “readers”.

TRMT6, TRMT61A, TRMT10C, and YTHDF1 were shown to be important regulators of m1A. To explore the latent biological function of m1A-related regulatory genes in HCC pathogenesis, enriched gene sets for these four genes were used to perform GSEA. GSEA suggested that increased TRMT6 expression was involved in various biological functions in the nucleus. Upregulation of TRMT10C was associated with cell division and the MYC pathway (Fig. 7a,b). High TRMT61A expression was related to protein metabolism, and high YTHDF1 expression has been associated with mitosis (Fig. 7c,d). Finally, to better understand the overview, a schematic representation shows the “writers”, “erasers”, and “readers” of m1A (Fig. 7e). m1A is deposited on mRNAs by “writers”, removed by “erasers”, and interpreted by “readers”.

## Discussion

As an emerging research hotspot, m1A RNA methylation has been associated with multiple fundamental biological processes, such as protein translation and RNA metabolism^16^. A recent study demonstrated that m1A localizes near start codons of apparent regulatory importance to increase translation efficiency and protein levels^11^. Another research suggested that m1A plays a dynamic role in responses to various conditions of stress and other physiological events^17^. Mounting evidence also supports that m1A-related regulatory gene alterations are closely connected to multiple diseases including esophageal carcinoma, colorectal adenocarcinoma, and bladder urothelial carcinoma^22,23^. However, the role of m1A RNA methylation in the development and prognosis of HCC remains unclear. For the first time, our study investigated alterations to m1A-related regulatory genes in HCC and their correlation with clinicopathological features by exploring the TCGA-LIHC database. mRNA expression and the prognostic values for these m1A-related regulatory genes in HCC patients were also explored, along with the seeking of the biological functions and signaling pathways that contribute to our understanding of how m1A RNA modifications function in HCC. In the end, a validation dataset ICGC-LIHC-JP confirmed the relationship between the expression of these four genes and patient survival, supporting the potential for m1A-related regulatory genes to be used as therapeutically important HCC biomarkers.

In this TCGA-LIHC cohort, sequencing data revealed a high frequency of CNV in m1A-related regulatory genes. Such alterations to m1A-related regulatory genes may play a significant role in HCC tumorigenesis, since gene mutations and CNV can result in phenotypic changes tightly linked to oncogenesis. In addition, we evaluated the correlation between m1A-related regulator genetic alterations and clinicopathological features and found that CNV was positively associated with elevated cancer stages. Particularly, we found that TP53 alterations were prominently associated with mutations in m1A-related regulatory genes. TP53 is a common tumour suppressor gene mutated in cancers, and the tumour suppressor protein p53 encoded by TP53 is usually absent in a variety of tumour types^29–31^. Previous studies have shown that TP53 mutations are prognosticators of poor outcomes in HCC^32^. Further research into the molecular relationships between TP53 and m1A may improve our understanding of HCC.

To understand the connection between m1A-related regulatory gene CNV on mRNA expression, 315 HCC samples from TCGA were analyzed. The increased copy number of nine m1A-related regulatory genes was significantly linked with their high mRNA expression, while deletions led to decreased mRNA expression. Cluster analysis showed that m1A-related regulatory gene expression positively correlated with high TNM stage, and this correlation was verified in a subsequent analysis. We also showed that the expression of 10 m1A-related regulatory genes could be used to assess HCC patient risk, especially TRMT6, TRMT61A, TRMT10C, and YTHDF1. This result suggested that the expression of m1A-related regulatory genes could be useful prognostic markers for HCC.

The validation dataset ICGC-LIHC-JP confirmed that the genes TRMT6, TRMT61A, TRMT10C, and YTHDF1 had good risk prediction value for HCC survival, and elevated TRMT6 gene expression was associated with poor prognosis. TRMT6 and TRMT61A form a methyltransferase complex and catalyze the methylation of the N1 position on adenosine residues in mRNA. Prior studies have shown that deletion of the TRMT6/61 complex can reduce glioma cell proliferation and increase cell death^33^. Additional studies showed that TRMT6 frameshift mutations could potentially contribute to colorectal cancer^34^. In our study, GSEA suggests that the upregulation of TRMT6 is related to various biological processes and supports TRMT6 as a potential prognostic biomarker for HCC.

Our study revealed that the expression of YTHDF1 was obviously correlated with TRMT6. YTHDF1, an important m1A modified RNA binding protein, has been identified as “reader” in m6A modification in prior studies. The mechanism of m6A “reader” YTHDF1 in cancer has been extensively researched. A recent study using multi-omics analysis reported that YTHDF1 promotes ovarian cancer occurrence and development through binding to m6A-modified EIF3C mRNA and consequently enhancing the EIF3C translation, which EIF3C is a subunit of the protein translation initiation factor EIF3^35^. Another research found that anti-tumour immunity in dendritic cells (DCs) was regulated by the m6A-binding protein YTHDF1, and the deficiency of YTHDF1 enhanced the cross-presentation of tumour antigens on DCs by decreasing the translation of lysosomal proteases in DCs^36^. These findings indicated that YTHDF1 is a novel target in the process of tumorigenesis and worthy of further mechanistic exploration in HCC.

In our study, GSEA results offered the biological function of m1A-related regulatory genes involved in HCC. Overall, the expression of m1A-related regulatory genes was related to several key biological processes and oncogenic characteristics, including cell cycle, mitosis, protein metabolism, and the MYC pathway, which provide clues to its contribution to HCC pathogenesis. Interestingly, a study conducted by Y He et al. found that m5C-related regulatory genes involved in cell cycle regulation and mitosis in HCC, which had a consistence with our results^14^. MYC is a transcription factor that regulates cell differentiation and proliferation through mechanisms such as the transcriptional amplification of target genes^37^. Studies have shown that MYC plays an important role in aerobic respiration and mitochondrial function, along with regulating energy metabolism through the MYC/ERRα signaling pathway^38^. Moreover, it has been demonstrated that MYC activation is a major genetic event towards liver cancer development, a recent study linked liver cancer formation with amino acid transporters regulated by MYC and the activation of the mTORC1 signaling pathway^39^. This justifies further exploration of the interplay between m1A and the MYC pathway in the progression of liver cancer.

In researching the possible carcinogenic mechanism of m1A-related regulatory genes in HCC, PI3K/Akt signaling pathway was found to be the most important item through KEGG analysis. The PI3K/Akt signaling pathway has been reported to involve in proliferation and anti-apoptosis process in HCC^40,41^, which pointed out a theoretical basis for further mechanism research on m1A-related regulatory genes. Following, relevant validations via cell and animal experiment are crucial to carry out to reveal how m1A-related regulatory genes regulate PI3K/Akt pathway in HCC.

In summary, we identified for the first time the alterations of m1A-related regulatory genes in HCC, and found a clear relationship with clinicopathological features and prognosis. TRMT6, TRMT61A, TRMT10C, and YTHDF1 effectively predicted HCC patient survival and contributed to important biological processes. TRMT6 showed promise as a potential prognostic biomarker for HCC. The MYC pathway and the PI3K/Akt signaling pathway may be involved in regulating m1A in HCC cells. To further clarify the molecular mechanism of m1A mRNA modification in HCC development, future studies in vivo and in vitro will aid in confirming our findings and molecular understanding.

## Material and methods

### Selection of m1A-related regulators

The 10 m1A-related regulators were determined through retrieving published literatures. According to previously reported researches, it’s found that TRMT10C, TRMT61B, TRMT6, and TRMT61A were identified as “writers”^42,43^; ALKBH1 and ALKBH3 were classified as “erasers”^17,19,44^; YTHDF1, YTHDF2, YTHDF3, and YTHDC1 were regarded as “readers”^18^.

### The Cancer Genome Atlas (TCGA) dataset

All patient clinical data, mutations, CNV, and mRNA expression data were retrieved and downloaded from the TCGA website (https://gdc.cancer.gov) by the TCGA-assembler in September 2019, as described previously^45^. Within TCGA-LIHC database, the transcriptome data for 371 HCC samples were downloaded as TPM (transcripts per million) and FPKM (fragments per kilobase per million mapped reads) for analysis. A total of 364 HCC samples were identified for SNV studies, which were downloaded as level3 data. For CNV data, there were 376 HCC samples. In addition, we downloaded 378 HCC samples containing clinical information to analysis the correlation between m1A-related regulatory gene alterations and clinicopathological features. The exclusion criteria were as follows: (1) HCC samples without CNV data and transcriptome data; (2) HCC samples with incomplete clinical information; (3) the survival time of HCC samples was less than 90 days. After the non-eligible cases were removed, there were 315 samples used for survival analysis.

### International Cancer Genome Consortium (ICGC) dataset

The validation data set ICGC was used to validate the results of four m1A-related regulatory genes as important HCC target molecules, which was downloaded from the ICGC website^46^ (https://dcc.icgc.org). The dataset ICGC-LIHC-JP was adopted and processed the same way as the TCGA cohort. After excluding HCC cases with incomplete clinical information and survival time less than 90 days, these additional validate studies were performed on 212 HCC cases.

### Bioinformatic analysis

GSEA was conducted to explore the latent biological function of m1A-related regulatory genes TRMT6, TRMT61A, TRMT10C, and YTHDF1 in HCC pathogenesis, as described previously^47^. We analyzed the enrichment gene sets associated with mRNA expression levels of these four genes in the TCGA dataset. GSEA v3.0 software (https://www.broad.mit.edu/gsea/) was used for analysis as described previously. A false discovery rate (FDR) < 0.25 and a nominal *P* < 0.05 were chosen as the significance cutoff criteria. Next, we extracted proteomic data from RPPA through the cBioPortal database, as explained previously^23^. Fold change (FC) > 1 and *p* value < 0.05 were chosen as the cutoff point to distinguish differentially expressed proteins. KEGG pathway analyses were performed by R package “clusterProfiler” (*p*_adjust_ < 0.05). Only the top 10 KEGG pathway terms were presented.

### The least absolute shrinkage and selection operator (LASSO)

The LASSO regression analysis is an accepted method for the reduction in high dimensional data, as described previously^13^. In this study, LASSO regression analysis was performed by R package “glmnet” to filtrate most powerful prognostic markers of m1A-related regulatory genes, and disease risk prediction score was established in HCC patients.

### Development of risk score

The risk score of the m1A-related regulatory genes was calculated based on multivariate Cox regression model as follows:

$$ {\text{Risk}}\,{\text{score}} = \mathop \sum \limits_{i = 1}^{N} Coefi \times xi $$

which $Coefi$ is the coefficient of Cox-regression model, and $xi$ is the expression levels of m1A-related regulatory genes. The median risk score was used to stratify the HCC patients. According to the median risk, HCC samples were divided into high-risk and low-risk groups. Kaplan–Meier survival analysis were performed to evaluate the survival probability in high-risk and low-risk groups using R package “survival”. The prognostic efficiency of the risk score was assessed by calculating the areas under the curve (AUC) of the time-dependent receiver operating characteristic (ROC) curve by R package “TimeROC”.

### Statistical analysis

All statistical analyses were performed using R version 3.4.1 (https://www.r-project.org/), SPSS 21.0 (SPSS Inc., Chicago, IL) and Prism 7.0 (GraphPad Software Inc., La Jolla, CA). Chi-square test was used to analyze the relationship between m1A-related regulatory genes alterations and clinicopathological characteristics. Statistical *t* tests were used to analyze m1A-related regulator gene expression against CNV patterns and clinical stages. Spearman correlation analysis was adopted to understand the relevance among the ten m1A-related regulatory genes. The prognostic values of m1A-related regulatory genes in HCC patients were analyzed by Kaplan–Meier curve, log-rank test, and Cox regression analysis. The prediction efficiency of risk score was tested with time-dependent ROC curves. The AUC were calculated to evaluate the property of the models. *P* values < 0.05 were considered statistically significant.

## Supplementary information

Supplementary file1
