# Supplementary material for: Gene signatures and prognostic values of m1A-related regulatory genes in hepatocellular carcinoma
Source: Sci Rep. 2020 Sep 15;10:15083. doi: 10.1038/s41598-020-72178-1 (PMC7492257; doi:10.1038/s41598-020-72178-1)
Supplement: Supplementary file 1 — Supplementary file1 [file 41598_2020_72178_MOESM1_ESM.pdf]

Gene signatures and prognostic values of m1A-related regulatory genes in hepatocellular carcinoma

Qingmiao Shi[1,2†], Chen Xue[1,2†], Xin Yuan[1,2†], Yuting He[1,2*], Zujiang Yu[1,2*]

[1]Gene Hospital of Henan Province, Precision Medicine Center, the First Affiliated Hospital of Zhengzhou University, Zhengzhou, Henan 450052, P.R. China

[2]Department of Infectious Diseases, the First Affiliated Hospital of Zhengzhou University, Zhengzhou, Henan 450052, P.R. China

†These authors contributed equally to this work.

*** Correspondence Author:**

Yuting He, MD, the First Affiliated Hospital of Zhengzhou University, Zhengzhou, Henan, 450052; Tel / fax: 0086-371-67967126; Email: fccheyt1@zzu.edu.cn

Zujiang Yu, Pro. & Ph.D, the First Affiliated Hospital of Zhengzhou University, Zhengzhou, Henan, 450052; Tel / fax: 0086-371-66271133; Email: yzjzzu@126.com

Supplementary Material

SUPPLEMENTARY TABLES

Supplementary Table 1. Statistics on the number of m1A-related regulatory gene mutations in LIHC samples.

	writer				reader				eraser	
Sample ID	TRMT10C	TRMT61B	TRMT6	TRMT61A	YTHDF1	YTHDF2	YTHDF3	YTHDC1	ALKBH1	ALKBH3
TCGA-2Y-A9H9				1			1	1		
TCGA-4R-AA8I					1					
TCGA-5C-AAPD			1							
TCGA-BC-A10Y							1			
TCGA-CC-5258		1								
TCGA-CC-5264		1			2					
TCGA-DD-A115	1									
TCGA-DD-A116							1			
TCGA-DD-A11C										1
TCGA-DD-A1EE	1				1					
TCGA-DD-AACI								1		
TCGA-DD-AADO										
TCGA-DD-AAE8								1		
TCGA-DD-AAEH										
TCGA-EP-A12J	1									
TCGA-FV-A23B										
TCGA-G3-AAV0	1									

TCGA-NI-A8LF						1				
TCGA-O8-A75V				1						
TCGA-XR-A8TG								1		

Supplementary Table 2. Functional changes of m1A-related regulatory gene mutations in LIHC samples.

Tumor Sample Barcode	Missense Mutation	Nonsense Mutation	Splice Site Mutation	total
TCGA-CC-5264	1	1	0	2
TCGA-DD-A1EE	2	0	0	2
TCGA-2Y-A9H9	1	0	0	1
TCGA-4R-AA8I	1	0	0	1
TCGA-5C-AAPD	1	0	0	1
TCGA-BC-A10Y	1	0	0	1
TCGA-CC-5258	0	0	1	1
TCGA-DD-A115	1	0	0	1
TCGA-DD-A11C	1	0	0	1
TCGA-DD-AAE8	1	0	0	1
TCGA-EP-A12J	1	0	0	1
TCGA-XR-A8TG	1	0	0	1

Supplementary Table 3. Functional annotation of seven m1A-related regulatory genes in LIHC samples.

Genes	Missense Mutation	Nonsense Mutation	Splice Site Mutation	total	Mutated Samples	Altered Samples
YTHDF1	3	1	0	4	3	3
TRMT10C	3	0	0	3	3	3
YTHDC1	3	0	0	3	3	3
ALKBH3	1	0	0	1	1	1
TRMT6	1	0	0	1	1	1
TRMT61B	0	0	1	1	1	1
YTHDF3	1	0	0	1	1	1

Supplementary Table 4. CNV statistics of m1A-related regulatory genes in LIHC samples.

Function	Genes	Diploid	Amplification	Deletion	CNV sum	amplification%	Deletion%	Percentage
writer	TRMT10C	328	36	13	49	73.47%	26.53%	13.00%
	TRMT61B	323	44	10	54	81.48%	18.52%	14.32%
	TRMT6	263	94	23	117	80.34%	19.66%	30.79%
	TRMT61A	254	17	106	123	13.82%	86.18%	32.63%
reader	YTHDF1	275	98	4	102	96.08%	3.92%	27.06%
	YTHDF2	253	10	114	124	8.06%	91.94%	32.89%
	YTHDF3	174	184	20	204	90.20%	9.80%	53.97%
	YTHDC1	253	13	119	132	9.85%	90.15%	34.29%
eraser	ALKBH1	268	15	96	111	13.51%	86.49%	29.29%
	ALKBH3	327	23	32	55	41.82%	58.18%	14.40%

Supplementary Table 5. The relationship between m1A regulated gene expression levels and patient prognosis by univariate Cox regression analysis

Genes	beta	HR (95% CI)	wald.test	*P*	CNV sig
TRMT6	0.24	1.3 (1.2-1.4)	27	1.80E-07	yes
YTHDF2	0.12	1.1 (1.1-1.2)	24	9.50E-07	yes
TRMT10C	0.084	1.1 (1-1.1)	17	3.40E-05	yes
YTHDF1	0.074	1.1 (1-1.1)	16	6.80E-05	yes
TRMT61A	0.078	1.1 (1-1.1)	11	0.00099	yes
ALKBH1	0.34	1.4 (1.1-1.8)	8.5	0.0035	yes
TRMT61B	0.14	1.2 (1-1.3)	6.7	0.0095	yes
YTHDC1	0.068	1.1 (0.99-1.2)	2.9	0.088	yes
ALKBH3	0.052	1.1 (0.96-1.1)	1.4	0.25	no
YTHDF3	0.015	1 (0.97-1.1)	0.47	0.5	yes
